# Traditional Chinese Medicine and Aging Intervention

**DOI:** 10.14336/AD.2017.1002

**Published:** 2017-12-01

**Authors:** Haiping Zhao, Yumin Luo

**Affiliations:** ^1^Cerebrovascular Diseases Research Institute, Xuanwu Hospital of Capital Medical University, Beijing, China; ^2^Center of Stroke, Beijing Institute for Brain Disorders, Beijing, China; ^3^Beijing Key Laboratory of Translational Medicine for Cerebrovascular Diseases, Beijing, China

Aging is an irreversible dynamic process that affects all humans. From ancient times, humankind has been interested in keeping young. Nowadays, the significance of anti-aging has changed from merely prolonging lifespan to improving healthspan. Traditional Chinese herbal medicine has a long history in Asian countries, which have antiaging properties and could intervene aging-associated disorders. The effectiveness of traditional Chinese medicine (TCM) relies on its large variety of naturally active chemicals, multiple targets for therapy, and diversity of treatment approaches. We have just launched a special issue entitled “Traditional Chinese Medicine and Aging Intervention” which contains ten high-quality review articles of different kinds of herbs that deploys plant species, characteristics, active ingredients, pharmacological effects and mechanisms of action of anti-aging and aging-related disease.

In this special issue, four drugs that are known to invigorate Qi and strengthen Yang according to TCM theory are discussed. *Ginseng* (Renshen) and *Astragalus membranaceus* (Huangqi) are two of the most highly regarded Chinese herbalism for invigorating Qi. Yang *et al.* extensively describe the plant species, characteristics, processing and active part and ingredients of *Ginseng*. Recent phytochemical and pharmacological studies have discovered a variety of potent compounds in all parts of the ginseng plant including ginsenosides, alkaloids, phenolics, phytosterol, carbohydrates, polypeptides, ginseng oils, amino acids, nitrogenous substances, vitamins, minerals, and certain enzymes. Ginsenosides are the major bioactive metabolites. They can increase lifespan, regulate the function of multiple organ systems including cardiovascular, nervous, and cutaneous systems through antioxidant and antiinflammatory properties. A proprietary extract from North American *Ginseng*, CVT-E002, has been shown to extend the lifespan of infant and juvenile mice with leukemia in a dose-dependent manner [1]. The next review by Liu *et al*., confers the major components of *Astragalus membranaceus* such as saponins, flavonoids, and polysaccharides. The experimental and clinical studies have demonstrated the immunoregulatory, antioxidant, hypolipidemic, antihyperglycemic, hepatoprotective, expectorant, and diuretic effects of *Astragalus membranaceus*. These properties likely contribute to its beneficial effects such as lifespan extension, slowing down of vascular and brain aging, and anti-cancer activity. Among these bioactive ingredients, TA-65, a proprietary extract of the dried root, has been found to be associated with significant anti-aging effects through the lengthening of telomeres in cells [2]. *Ganoderma lucidum* (Lingzhi), a white-rot fungus, considered as an elixir, has been around for thousands of years. Wang *et al*., describe in detail the components of ethanol, aqueous, mycelia, and water-soluble extracts of *G. lucidum*, including polysaccharides, triterpenes, and peptidoglycans. Ganodermasides, *Ganoderma lucidum* peptide, *Ganoderma* polysaccharide peptide, total *G. lucidum* triterpenes and Ganoderic acid C1 could exert lifespan elongation or related activities [3]. Besides these direct anti-aging effects, the immunomodulatory, antioxidant, neuroprotective, and anti-tumor effects have been suggested. Also, as a 'Yang-invigorating' tonic herb, *H. cistanches* (Roucongrong) has been used for chronic renal disease, impotence, female infertility, morbid leucorrhea, profuse menorrhagia and senile constipation. *Herba cistanches* is widely accepted and has earned the honor of “*Ginseng* in the deserts.” Wang *et al*. also elaborate on the components of ethanol, aqueous, and methanol extracts of *H. cistanches*, including echinacoside, acteoside, isoacteoside, and poly-saccharides. The extraction of *H. cistanches* with direct lifespan elongation effects or potential anti-aging properties mainly includes the ethanolic extract of H. cistanches, Herba cistanches aqueous extract and Methanol extract. The review summarizes the comprehensive information on *Herba cistanches* covering many aspects of botany, traditional uses, phytochemistry, pharmacology, and its application to clinical practice on antiaging [4].

In this special issue, two kinds of drugs that nourish Yin are also described. These include *Lycium barbarum* (Gouqi) and *Dendrobium* (Shihu), which have been used as traditional medicinal herbs and food supplements for thousands of years. Chen *et al*., have summarized the main components of *L. barbarum,* which contains abundant polysaccharides, betaine, phenolics, carotenoids, cerebroside, 2-O-β-d-glucopyranosyl-l-ascorbic acid, β-sitosterol, flavonoids, and vitamins. However, *L. barbarum* polysaccharides are the primary active components of *L. barbarum*, which are reported to mediate significant anti-aging effect through antioxidant, immunoregulative, anti-apoptotic activities and to reduce DNA damage [5].* Dendrobium* is one of the earliest recorded orchids in ancient China, and Chinese use *Dendrobium* tonic for longevity. *Dendrobium* tonic contains astringent, analgesic, antipyretic, and anti-inflammatory substances, and are traditionally used as medicinal herbs in the treatment of a variety of disorders, such as nourishing the stomach, enhancing production of body fluids or nourishing Yin. The review by Cakova *et al*., is focused on anticancer, anti-diabetic, neuroprotective and immunomodulating activities of different *Dendrobium* species and their constituents, to report their high promise for treating age-related pathologies [6].

In addition, another two drugs including *Rhizoma coptidis* (Huanglian) and *Scutellaria baicalensis* (Huangqin) are two of the strongest herbs to clear heat, dry dampness, and eliminate toxins according to the TCM theory. *Rhizoma coptidis* has been used to clear heat and to dry dampness in the stomach or intestines, which manifests as diarrhea or dysenteric disorder. Xu *et al*., summarized that the evidence supports the possibility that *R. coptidis*, in particular, berberine, is a promising anti-aging natural product, and has pharmaceutical potential in combating aging-related diseases. The mechanism of these effects involves anti-oxidation, activation of AMPK signaling and its downstream targets including mTOR/rpS6, Sirtuin1/forkhead box transcription factor O3 (FOXO3), nuclear factor erythroid-2 related factor-2 (Nrf2), nicotinamide adenine dinucleotide (NAD+) and nuclear factor-κB (NF-κB) pathways [7]. And unlike *Rhizoma coptidis*, *Scutellaria baicalensis* acts on the lungs, such as to clear away heat, reduce phlegm, and relieve cough. Chen *et al*., indicated that Baicalin and its aglycon baicalein are the principal components among other flavonoid derivatives in the roots of *Scutellaria baicalensis*. Abundant scientific evidence shows that the neuronal protective effects of baicalin and baicalein against cerebral ischemia are related to anti-oxidant, anti-apoptotic, anti-inflammatory and anti-excitotoxicity effects, protection of the mitochondria, induction of the expression of protective factors in neurons, increased adult neurogenesis, and other factors [8].

Moreover, two herbs promoting blood circulation to dissipate blood stasis and dredging collaterals to relieve pain, *Panax notoginseng* (Sanqi) and *Ginkgo biloba leaves* (Yinxingye) were introduced. *Panax notoginseng*, a species of the genus *Panax*, has been called the "miracle root for the preservation of life," and has been extensively employed in China to treat microcirculatory disturbances, inflammation, trauma, internal and external bleeding due to injury, and as a tonic. Zhao introduces ethno-pharmacology, phytochemistry, and pharmacology of *Panax notoginseng*, as well as its pharmacological function on lifespan extension, anti-vascular aging, anti-brain aging, and anti-cancer properties [9]. Furthermore, *G. biloba leaves* are widely used in various degenerative diseases such as cerebrovascular disease, Alzheimer’s disease, macroangiopathy and more. The review by Zuo summarized several pharmacological mechanisms of *Ginkgo biloba leaves* extract including its function as an antioxidant, prevention of mitochondrial dysfunction, and effect on apoptosis. And the review also described some clinical applications of *G. biloba leaves* extract, such as its effect on neuro- and cardiovascular protection, and anticancer properties [10].

In this special issue, we present a unique group of papers on “Traditional Chinese Medicine and Aging Intervention” for readers. TCM drug discovery is proceeding at a faster pace with the aid of new advanced technologies. The mechanistic insights to be uncovered will help to unleash the pharmacological potential embedded in the traditional principles of TCM. We look forward to publishing more high-quality papers in the coming year contributing to advances in the pharmacology of anti-aging TCM herbs covering all aspects from basic, translational to clinical pharmacology. We hope that the readers will enjoy this special issue. We would look forward to receiving your suggestions, comments, and manuscript contribution for future special issues.

## References

[1] Yang Y, Ren CH, Zhang Y, Wu XD (2017). Ginseng: an nonnegligible natural remedy for healthy aging. Aging Dis, 8: 708-720. 10.14336/AD.2017.0707 PMC5758347 29344412

[2] Liu P, Zhao HP, Luo YM (2017). Anti-aging implications of Astragalus Membranaceus (Huangqi): a well-known chinese tonic. Aging Dis, 8: 868-886. 10.14336/AD.2017.0816 PMC5758356 29344421

[3] Wang J, Cao B, Zhao HP, Feng J (2017). Emerging roles of Ganoderma Lucidum in anti-aging. Aging Dis, 8: 691-707 10.14336/AD.2017.0410 PMC5758346 29344411

[4] Wang NQ, Ji SZ, Zhang H, Mei SS, Qiao LM, Jin XL (2017). Herba Cistanches: anti-aging. Aging Dis, 8: 740-759. 10.14336/AD.2017.0720 PMC5758349 29344414

[5] Gao YJ, Wei YF, Wang YQ, Gao F, Chen ZG (2017). Lycium Barbarum: a traditional chinese herb and a promising anti-aging agent. Aging Dis, 8: 778-791. 10.14336/AD.2017.0725 PMC5758351 29344416

[6] Cakova V, Bonté F, Lobstein A (2017). Dendrobium: sources of active ingredients to treat age-related pathologies. Aging Dis, 8: 827-849. 10.14336/AD.2017.0214 PMC5758354 29344419

[7] Xu ZF, Feng W, Shen Q, Yu NN, Yu K, Wang SJ, Chen ZG, Shioda S, Guo Y (2017). Rhizoma Coptidis and berberine as a natural drug to combat aging and aging-related diseases via anti-oxidation and AMPK activation. Aging Dis, 8: 760-777. 10.14336/AD.2016.0620 PMC5758350 29344415

[8] Liang W, Huang XB, Chen WQ (2017). The effects of Baicalin and Baicalein on cerebral ischemia: a review. Aging Dis, 8: 850-867. 10.14336/AD.2017.0829 PMC5758355 29344420

[9] Zhao HP, Han ZP, Li GW, Zhang SJ, Luo YM (2017). Therapeutic potential and cellular mechanisms of panax notoginseng on prevention of aging and cell senescence-associated diseases. Aging Dis, 8: 721-739. 10.14336/AD.2017.0724 PMC5758348 29344413

[10] Zuo W, Yan F, Zhang B, Li JT, Mei D (2017). Advances in the studies of Ginkgo Biloba leaves extract on aging-related diseases. Aging Dis, 8: 812-826. 10.14336/AD.2017.0615 PMC5758353 29344418

